# Experimental Pretreatment with Chlorogenic Acid Prevents Transient Ischemia-Induced Cognitive Decline and Neuronal Damage in the Hippocampus through Anti-Oxidative and Anti-Inflammatory Effects

**DOI:** 10.3390/molecules25163578

**Published:** 2020-08-06

**Authors:** Tae-Kyeong Lee, Il-Jun Kang, Bora Kim, Hye Jin Sim, Dae- Won Kim, Ji Hyeon Ahn, Jae-Chul Lee, Sungwoo Ryoo, Myoung Cheol Shin, Jun Hwi Cho, Young-Myeong Kim, Joon Ha Park, Soo Young Choi, Moo-Ho Won

**Affiliations:** 1 Department of Biomedical Science and Research Institute for Bioscience and Biotechnology, Hallym University, Chuncheon, Gangwon 24252, Korea; tk-lee@hallym.ac.kr (T.-K.L.); jh-ahn@hallym.ac.kr (J.H.A.); 2 Department of Food Science and Nutrition, Hallym University, Chuncheon, Gsangwon 24252, Korea; ijkang@hallym.ac.kr; 3 Department of Neurobiology, School of Medicine, Kangwon National University, Chuncheon, Gangwon 24341, Korea; nbrkim17@gmail.com (B.K.); janny20@kangwon.ac.kr (H.J.S.); anajclee@kangwon.ac.kr (J.-C.L.); 4 Department of Biochemistry and Molecular Biology, and Research Institute of Oral Sciences, College of Dentistry, Gangnung-Wonju National University, Gangneung, Gangwon 25457 Korea; kimdw@gwnu.ac.kr; 5 Department of Biological Sciences, College of Natural Sciences, Kangwon National University, Chuncheon, Gangwon 24341, Korea; ryoosw08@kangwon.ac.kr; 6 Department of Emergency Medicine, School of Medicine, Kangwon National University, Chuncheon, Gangwon 24341, Korea; dr10126@naver.com (M.C.S.); cjhemd@kangwon.ac.kr (J.H.C.); 7 Department of Molecular and Cellular Biochemistry, School of Medicine, Kangwon National University, Chuncheon, Gangwon 24341, Korea; ymkim@kangwon.ac.kr; 8 Department of Anatomy, College of Oriental Medicine, Dongguk University-Gyeongju, Gyeongju, Gyeongbuk, 38066, Korea; jh-park@dongguk.ac.kr

**Keywords:** anti-inflammation, antioxidation, chlorogenic acid, hippocampal neurons, neuroprotection, transient forebrain ischemia

## Abstract

Chlorogenic acid (CGA), an ester of caffeic acid and quinic acid, is among the phenolic acid compounds which can be naturally found in green coffee extract and tea. CGA has been studied since it displays significant pharmacological properties. The aim of this study was to investigate the effects of CGA on cognitive function and neuroprotection including its mechanisms in the hippocampus following transient forebrain ischemia in gerbils. Memory and learning following the ischemia was investigated by eight-arm radial maze and passive avoidance tests. Neuroprotection was examined by immunohistochemistry for neuronal nuclei-specific protein and Fluoro-Jade B histofluorescence staining. For mechanisms of the neuroprotection, alterations in copper, zinc-superoxide dismutase (SOD1), SOD2 as antioxidant enzymes, dihydroethidium and 4-hydroxy-2-nonenal as indicators for oxidative stress, and anti-inflammatory cytokines (interleukin (IL)-4 and IL-13) and pro-inflammatory cytokines (tumor necrosis factor α (TNF-α) and IL-2) were examined by Western blotting and/or immunohistochemistry. As a result, pretreatment with 30 mg/kg CGA attenuated cognitive impairment and displayed a neuroprotective effect against transient forebrain ischemia (TFI). In Western blotting, the expression levels of SOD2 and IL-4 were increased due to pretreatment with CGA and, furthermore, 4-HNE production and IL-4 expressions were inhibited by CGA pretreatment. Additionally, pretreated CGA enhanced antioxidant enzymes and anti-inflammatory cytokines and, in contrast, attenuated oxidative stress and pro-inflammatory cytokine expression. Based on these results, we suggest that CGA can be a useful neuroprotective material against ischemia-reperfusion injury due to its antioxidant and anti-inflammatory efficacies.

## 1. Introduction

A brief period of ischemic condition in the brain causes ischemia-reperfusion injury, and it leads to selective neuronal loss/death in brain regions vulnerable to ischemia-reperfusion injury, such as the neocortex, hippocampus and striatum [1,2,3,4]. Transient forebrain ischemia (TFI) for 5 min causes death of pyramidal neurons in the hippocampal Cornu Ammonis 1 (CA1) region. The neuronal loss/death following 5 min TFI occurs from 4 or 5 days after TFI; thus, this phenomenon is termed as the “delayed neuronal death” [2,5].

Many studies on the mechanisms regarding to the delayed neuronal death following transient brain ischemia are constantly carried out. Among the mechanisms, oxidative stress due to the overproduction of reactive oxygen species (ROS) and inflammatory responses following infiltration of inflammatory cytokines have been strongly proposed [6,7,8,9]. Based on the above-mentioned mechanisms, neuroprotective materials against transient ischemic injury were investigated [10,11].

Chlorogenic acid (CGA), an ester formed from caffeic acid and L-quinic acid, is the major polyphenolic compound in coffee isolated from the leaves and fruits of dicotyledonous plants including *Coffea canephora* and *Coffea arabica* L. [12]. It has been reported that pretreatment with CGA attenuates neuronal loss in the cerebral cortex following transient focal brain ischemia induced by middle cerebral artery occlusion in rats [13]. Furthermore, the therapeutic administration of CGA attenuates cognitive and memory impairment and protects hippocampal neurons through its antioxidant and anti-apoptotic attributes in a rat model of transient global cerebral ischemia [14].

To the best of our knowledge, quantitative verifications of neuroprotective mechanisms by antioxidative and anti-inflammatory efficacies of CGA in the process of time after transient brain ischemia have been poorly conducted. Therefore, the objective of the present study was to examine whether the pretreatment of CGA protected pyramidal neurons in the hippocampal CA1 region from ischemic injury induced by TFI in gerbils, which are used as an excellent animal model of TFI [8,15,16]. Then, we investigated whether the protective mechanisms of CGA against the ischemic injury were related to antioxidative and anti-inflammatory roles by Western blotting and immunohistochemical analyses at 1 and 5 days after TFI.

## 2. Results

### 2.1. Cognitive Function

#### 2.1.1. Spatial Memory

**Figure 1:**
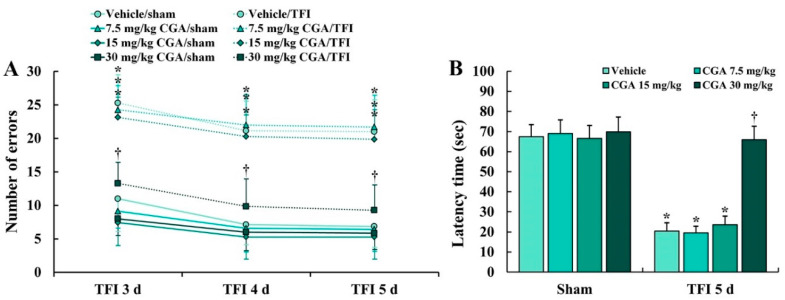
(**A**) Numbers of errors in 8-ARMT in the vehicle/sham, vehicle/ transient forebrain ischemia (TFI), chlorogenic acid (CGA) (7.5, 15 and 30 mg/kg)/sham and CGA/TFI groups at 3, 4 and 5 days after TFI. The numbers of the errors in the 30 mg/kg CGA/TFI group were remarkably reduced after TFI. (**B**) Latency time in the passive avoidance test (PAT) in the vehicle/sham, vehicle/TFI, CGA/sham and CGA/TFI groups at 5 days after TFI. In the 30 mg/kg CGA/TFI group, the latency time was significantly increased compared to that in the vehicle/TFI group. The bars indicate the means ± SEM (*n* = 7 in each group, * *p <* 0.05 vs. corresponding sham group, ^†^ *p <* 0.05 vs. vehicle/TFI group).

An 8 Arm radial maze test (8-ARMT) was conducted to investigate the difference in spatial memory between all experimental groups following TFI (Figure 1A). Numbers of errors in the 8-ARMT were not significantly different between all sham groups (groups pretreated with vehicle and CGA (7.5, 15 and 30 mg/kg) and subjected to sham TFI) at 3, 4 and 5 days after sham TFI (Figure 1A). However, in the vehicle/TFI group (group pretreated with vehicle and subjected to TFI) and 7.5 and 15 mg/kg CGA/TFI groups (groups pretreated with 7.5 and 15 mg/kg of CGA and subjected to TFI), the numbers of the errors were significantly increased after TFI (Figure 1A). On the other hand, the numbers of the errors in the 30 mg/kg CGA/TFI group were remarkably reduced after TFI (Figure 1A).

#### 2.1.2. Learning and Memory

A passive avoidance test (PAT) was carried out to examine the difference in learning and memory after TFI (Figure 1B). No significant difference in latency time in the PAT was found between all sham groups (Figure 1B). In the vehicle/TFI group, and 7.5 and 15 mg/kg CGA/TFI groups, the latency time was markedly shortened compared to that in the sham groups (Figure 1B). However, in the 30 mg/kg CGA/TFI group, the latency time was significantly increased compared to that in the vehicle/TFI group (Figure 1B).

### 2.2. Neuroprotection

#### 2.2.1. Neuronal Nucleus-Specific Protein (NeuN) Immunoreactive Neurons

**Figure 2:**
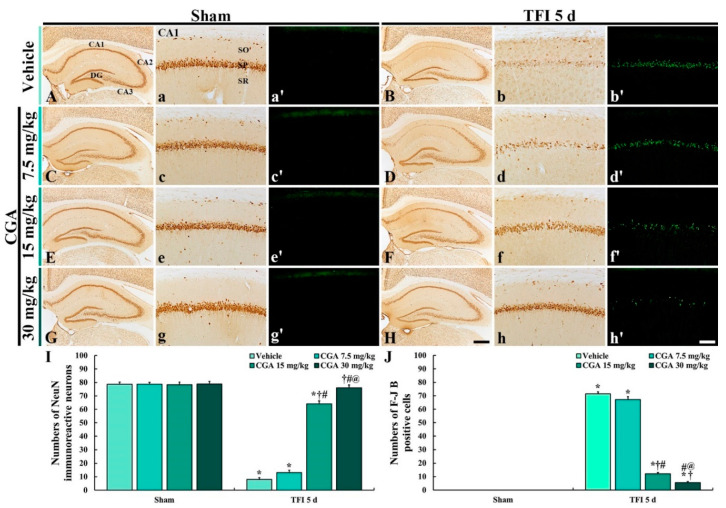
NeuN immunohistochemistry (**A**–**H**,**A**(**a**)–**H**(**h**)) and (**F**–**J**) B histofluorescence (**A**(**a’**)–**H**(**h’**)) in the hippocampus (**A**–**H**) and CA1 region (**A**(**a**)–**H**(**h**),**A**(**a’**)–**H**(**h’**)) of the vehicle/sham and vehicle/TFI (**A**(**a**,**a’**),**B**(**b**,**b’**)), 7.5 mg/kg CGA/sham and CGA/TFI (**C**(**c**,**c’**),**D**(**d**,**d’**)), 15 mg/kg CGA/sham and CGA /TFI (**E**(**e**,**e’**), **F**(**f**,**f’**) and 30 mg/kg CGA/sham and CGA/TFI (**G**(**g**,**g’**),**H**(**h**,**h’**)) groups at 5 days after TFI. NeuN^+^ neurons are markedly reduced in the stratum pyramidale (SP) of the CA1 region of the vehicle/TFI group. Numerous F-J B^+^ cells are observed in the SP of the vehicle/TFI, 7.5 mg/kg CGA/TFI and 15 mg/kg CGA/TFI groups. In the 30 mg/kg CGA/TFI group, numerous NeuN^+^ neurons and no F-J B^+^ cells are detected in the SP. DG, dentate gyrus; SO, stratum oriens; SP, stratum pyramidale; SR, stratum radiatum. Scale bars = 400 μm (**A**–**H**) 40 μm (**A**(**a**)–**H**(**h**),**A**(**a’**)–**H**(**h’**)). I and J: mean numbers of NeuN^+^ (**I**) and F-J B^+^ cells (**J**) in the SP of the CA1 region. The bars indicate the means ± SEM (*n* = 7 in each group, * *p <* 0.05 vs. the corresponding sham group, ^†^*p <* 0.05 vs. the vehicle/TFI group, ^#^ *p <* 0.05 vs. the 7.5 mg/kg CGA/TFI group, ^@^ *p <* 0.05 vs. the 15 mg/kg CGA/TFI group).

NeuN immunohistochemistry was conducted to examine intact neurons in the gerbil hippocampus at 5 days after TFI (Figure 2).

In all of the sham groups, NeuN (a marker for neurons) immunoreactive (NeuN^+^) neurons in the hippocampus were clearly observed, showing that NeuN^+^ neurons consisted of the stratum pyramidale (SP) (Figure 2A,C,E,G). In the vehicle/TFI group, NeuN^+^ neurons in the SP (called pyramidal cells or neurons) were significantly decreased in the CA1 region, not the CA2/3 region, compared to the vehicle/sham group (Figure 2B(b),I).

In the 7.5 mg/kg CGA/TFI group, the distribution pattern of the NeuN^+^ CA1 pyramidal neurons was similar to that in the vehicle/TFI group (Figure 2D(d),I). However, in the 15 mg/kg CGA/TFI group, the number of the NeuN^+^ CA1 pyramidal neurons was higher than that that in the vehicle/sham group (Figure 2F(f),I). In addition, the number of the NeuN^+^ CA1 pyramidal neurons in the 30 mg/kg CGA/TFI group was similarly to that in the vehicle/sham group (Figure 2H(h),I).

#### 2.2.2. Fluoro-Jade B (F-J B) Positive Cells

F-J B positive (F-J B^+^) cells are dead cells. Hence, F-J B histofluorescence was conducted to examine the loss of neurons or cells in the hippocampus at 5 days after TFI (Figure 2).

No F-J B^+^ cells were found in the CA1 region of all of the sham groups (Figure 2A(a’),C(c’),E(e’),G(g’). In contrast, numerous F-J B^+^ cells were found in the SP of the CA1 region of the vehicle/TFI group (Figure 2B(b’)).

In the 7.5 mg/kg CGA/TFI group, F-J B^+^ CA1 pyramidal cells were detected like those in the vehicle/TFI group (Figure 2D(d’),J). In the 15 mg/kg CGA/TFI group, many F-J B^+^ CA1 pyramidal cells were also found (Figure 2F(f)’,J). However, a few F-J B^+^ CA1 pyramidal cells were observed in the 30 mg/kg CGA/TFI (Figure 2H(h’),J).

Based on the results of NeuN immunohistochemistry and F-J B histofluorescence in all of the groups, the pretreatment with 30 mg/kg CGA displayed neuroprotection in the gerbil hippocampus after TFI. Therefore, we used 30 mg/kg of CGA for the following experiments.

### 2.3. Levels of 4-Hydroxy-2-Nonenal (4-HNE), SOD2, IL-2 and IL-4

**Figure 3:**
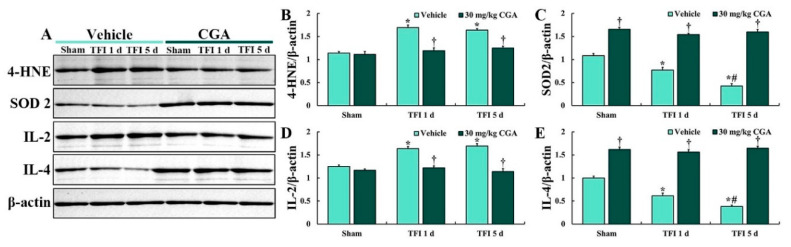
Representative Western blot images of 4-HNE, SOD2, IL-4 and IL-2 in the CA1 region (**A**) and densitometric analyses of them (**B**–**E**) in the vehicle/sham, vehicle/TFI, CGA/sham and CGA/TFI groups at 5 days after TFI. Levels of 4-HNE and IL-2 are increased in the vehicle/TFI group, but the 4-HNE and IL-2 levels in the GCA/TFI group are not altered after TFI. Levels of SOD2 and IL-2 are markedly decreased after TFI, but they are significantly higher in the CGA/sham and CGA/TFI groups than those in the vehicle/sham group days after TFI. The bars indicate the means ± SEM (*n* = 5 at each point in time after TFI, * *p <* 0.05 vs. the vehicle/sham group, ^†^ *p <* 0.05 vs. the corresponding time point vehicle group, ^#^ *p* < 0.05 vs. the pretime point vehicle group).

Western blotting of 4-HNE was conducted to examine the degree of lipid peroxidation in the CA1 region (Figure 3). In all of the sham groups, levels of 4-HNE were not significantly different between them (Figure 3A,B). In the vehicle/TFI group, the 4-HNE level was significantly increased at 1 day (about 148% vs. the vehicle/sham group) and 5 days (about 144% vs. the vehicle/sham group) after TFI (Figure 3A,B). In the CGA/sham group, the 4-HNE level was similar to that in the vehicle/sham group, and, in the CGA/TFI group, the level was maintained (about 105% vs. the vehicle/sham group at 1 day and 109% vs. the vehicle/sham at 5 days) after TFI.

Western analysis of SOD2 was conducted to examine the degree of endogenous SOD2 expression in the CA1 region (Figure 3). SOD2 level in the vehicle/TFI group was remarkably decreased at 1 day (about 71% vs. the vehicle/sham group) after TFI and more reduced 5 days (about 39% vs. the vehicle/sham group) after TFI (Figure 3A,C). In contrast, the SOD2 level of the CGA/sham group was significantly higher (about 153% vs. the vehicle/sham group) than that in the vehicle/sham group (Figure 3A,C). In addition, the SOD2 level of the CGA/TFI group was maintained (about 142% vs. the vehicle/sham group at 1 day and 147% vs. the vehicle/sham group at 5 days) after TFI (Figure 3A,C).

Western blotting for IL-2 was conducted to examine the degree of pro-inflammatory cytokine expression in the CA1 region (Figure 3). No significant difference in the IL-2 level was detected between all sham groups (Figure 3A,D). The IL-2 level in the vehicle/TFI group was remarkably increased at 1 day (about 131% vs. the vehicle/sham group) and 5 days (about 138% vs. the vehicle/sham group) after TFI (Figure 3A,D). However, the IL-2 level in the CGA/TFI group was lower (about 98% vs. the vehicle/sham group at 1 day) and (about 91% vs. the vehicle/sham group at 5 days after TFI) than that in the CGA/sham group (Figure 3A,D).

Western analysis of IL-4 was conducted to examine the degree of anti-inflammatory cytokine expression in the CA1 region (Figure 3). In the vehicle/TFI group, the IL-4 level was markedly reduced at 1 day (about 62% vs. the vehicle/sham group) and more reduced at 5 days (about 38% vs. the vehicle/sham group) after TFI (Figure 3A,E). In the CGA/sham group, the IL-4 level was markedly enhanced (about 162% vs. the vehicle/sham group) compared to that in the vehicle/sham group (Figure 3A,E). In the CGA/TFI group, the increased IL-4 level was sustained (about 157% vs. the vehicle/sham group at 1 day and 165% vs. the vehicle/sham group at 5 days) after TFI (Figure 3A,E).

### 2.4. Oxidative Stress in CA1 Pyramidal Cells

#### 2.4.1. Dihydroethidium (DHE) Fluorescence

**Figure 4:**
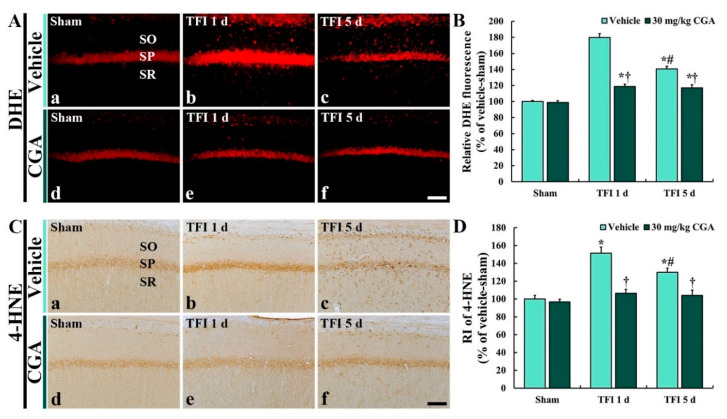
Dihydroethidium (DHE) histofluorescence (**A**) and 4-HNE immunohistochemistry (**B**) of the vehicle/sham (**A**(**a**),**C**(**a**)), CGA/sham (**A**(**d**),**C**(**d**)), vehicle/TFI (**A**(**b**,**c**),**C**(**c**,**d**)) and CGA/TFI (**A**(**e**,**f**),**C**(**e**,**f**)) groups at 1 and 5 days after TFI. DHE fluorescence and 4-HNE immunoreactivity in the vehicle/sham group are shown in CA1 pyramidal cells located in the SP. DHE fluorescence and 4-HNE immunoreactivity in CA1 pyramidal cells of the vehicle/TFI group are markedly enhanced after TFI, but CGA treatment apparently reduces their levels after TFI. SO, stratum oriens; SP, stratum pyramidale; SR, stratum radiatum. Scale bar = 40 μm. C and D: relative fluorescence of DHE (**C**) and relative immunoreactivity (RI) of 4-HNE (**D**) in the CA1 SP. The bars indicate the means ± SEM (*n* = 7 at each point in time, * *p <* 0.05 vs. the vehicle/sham group, ^†^ *p <* 0.05 vs. the corresponding time point vehicle group, ^#^ *p <* 0.05 vs. the pretime point vehicle/TFI group).

DHE histofluorescence staining in the CA1 region was conducted to examine the in situ production of superoxide anion (Figure 4). In the vehicle/sham group, DHE fluorescence was observed in the CA1 pyramidal cells located in the SP (Figure 4A(a)). However, DHE fluorescence in the CA1 pyramidal cells of the vehicle/TFI group was significantly enhanced (about 180% vs. the vehicle/sham group at 1 day and 141% vs. the vehicle/sham group at 5 days) after TFI (Figure 4A(b,c),B).

In the CGA/sham group, DHE fluorescence in the CA1 pyramidal cells was similar to that in the vehicle/sham group (Figure 4A(d)). DHE fluorescence in the CGA/TFI group was significantly lower (about 33.9% at 1 day and 16.6% at 5 days vs. the corresponding vehicle/TFI group) than that in the vehicle/TFI group (Figure 4A(e,f),B).

#### 2.4.2. 4-HNE Immunoreactivity

4-HNE immunohistochemistry in the CA1 region was performed to examine lipid peroxidation (Figure 4). In the vehicle/sham group, 4-HNE immunoreactivity was shown in the CA1 pyramidal cells (Figure 4C(a)). In the vehicle/TFI group, 4-HNE immunoreactivity in the CA1 pyramidal cells was significantly increased (about 152% at 1 day and 130% at 5 days vs. the vehicle/sham group) after TFI (Figure 4C(b),C(c),B).

In the CGA/sham group, 4-HNE immunoreactivity in the CA1 pyramidal cells was not different from that in the vehicle/sham group (Figure 4C(d),D). The 4-HNE immunoreactivity in the CGA/TFI group was markedly low (about 29.8% at 1 day and 19.9% at 5 days vs. the corresponding vehicle/TFI group) compared with that in the vehicle/TFI group (Figure 4C(e),C(f),D).

### 2.5. Antioxidant Enzyme Immunoreactivities in CA1 Pyramidal Cells

**Figure 5:**
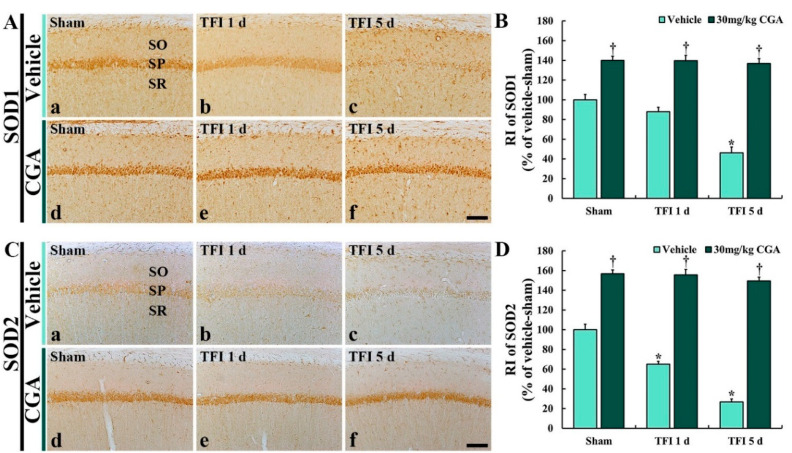
Immunohistochemistry for SOD1 (**A**) and SOD2 (**B**) of the vehicle/sham (**A**(**a**),**C**(**a**)), CGA/sham (**A**(**d**),**C**(**d**)), vehicle/TFI (**A**(**b**,**c**),**C**(**c**,**d**)) and CGA/TFI (**A**(**e**,**f**),**C**(**e**,**f**)) groups at 1 and 5 days after TFI. In the vehicle/sham group, SODs immunoreactivities are found in CA1 pyramidal cells, and SODs immunoreactivities in the vehicle/TFI group are decreased with time after TFI. However, SODs immunoreactivities in the CGA/sham group are significantly higher than those in the vehicle/sham group, and they are maintained after TFI. Scale bar = 40 μm. (**C**,**D**): RI of SOD1 (**C**) and SOD2, respectively, in the CA1 SP. The bars indicate the means ± SEM (*n* = 7 at each point in time, * *p <* 0.05 vs. the vehicle/sham group, ^†^ *p <* 0.05 vs. the corresponding time point vehicle group).

Changes in antioxidant enzyme immunoreactivities were examined by immunohistochemistry for SOD1 and SOD2 in the CA1 region (Figure 5).

#### 2.5.1. SOD1 Immunoreactivity

SOD1 immunoreactivity in the vehicle/sham group was easily detected in the CA1 pyramidal cells (Figure 5A(a)). In the vehicle/TFI group, SOD1 immunoreactivity in the CA1 pyramidal cells was decreased at 1 day (about 88% versus the vehicle/sham group) after TFI and more reduced at 5 days (about 46% versus the vehicle/sham group) after TFI (Figure 5A(b,c),B).

In the CGA/sham group, SOD1 immunoreactivity in the CA1 pyramidal cells was remarkably stronger (about 140% vs. the vehicle/sham group) than that in the vehicle/sham group (Figure 5A(d),B). In the CGA/TFI group, the increased SOD1 immunoreactivity was maintained (about 139% at 1 day and 137% at 5 days vs. the vehicle/sham group) after TFI (Figure 5A(e,f),B).

#### 2.5.2. SOD2 Immunoreactivity

In the vehicle/sham group, SOD2 immunoreactivity was obviously found in the CA1 pyramidal neurons (Figure 5C(a)). However, the SOD2 immunoreactivity in the vehicle/TFI group was markedly decreased at 1 day (about 65% vs. the vehicle/sham group) and more weakened at 5 days (about 27% vs. the vehicle/sham group) after TFI (Figure 5C(b,c),D).

On the other hand, in the CGA/sham group, the SOD2 immunoreactivity in the CA1 pyramidal cells was significantly increased (about 157% vs. the vehicle/sham group) compared to that in the vehicle/sham group (Figure 5C(d),D). In addition, the increased SOD2 immunoreactivity was sustained (about 156% at 1 day and 149% at 5 days vs. the vehicle/sham group) after TFI (Figure 5C(e,f),D).

### 2.6. Pro-Inflammatory Cytokine Immunoreactivities in CA1 Pyramidal Cells

**Figure 6:**
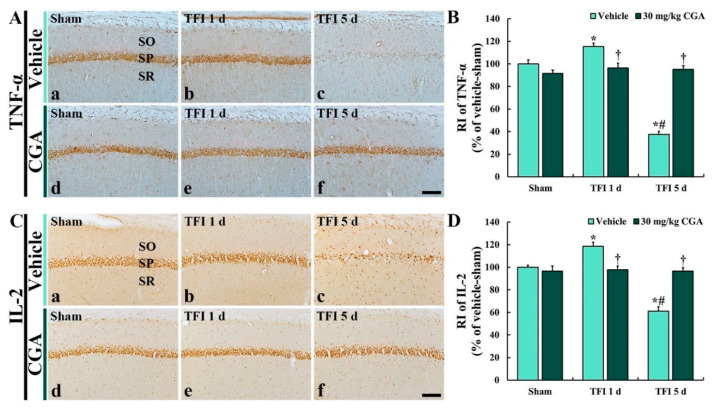
Immunohistochemistry for TNF-α (**A**) and IL-2 (**B**) of the vehicle/sham (**A**(**a**),**C**(**a**)), CGA/sham (**A**(**d**),**C**(**d**)), vehicle/TFI (**A**(**b**,**c**),**C**(**c**,**d**)) and CGA/TFI (**A**(**e**,**f**),**C**(**e**,**f**)) groups at 1 and 5 days after TFI. TNF-α and IL-2 immunoreactivities are basically detected in the CA1 pyramidal cells of the vehicle/sham group. However, TNF-α and IL-2 immunoreactivities in the vehicle/TFI group are dramatically altered after TFI. In the CGA/sham group, TNF-α and IL-2 immunoreactivities are similar to those in the sham groups, and these immunoreactivities are maintained after TFI. Scale bar = 40 μm, C and D: RI of TNF-α (**C**) and IL-2 (**D**), respectively, in theCA1 SP. The bars indicate the means ± SEM (*n* = 7 at each point in time, * *p <* 0.05 vs. the vehicle/sham group, ^†^ *p <* 0.05 vs. the corresponding time point vehicle group, ^#^ *p <* 0.05 vs. the pretime point vehicle group).

Changes in pro-inflammatory cytokine immunoreactivities were investigated by immunohistochemistry for TNF*-α* and IL-2 in the CA1 region (Figure 6).

#### 2.6.1. TNF-α Immunoreactivity

In the vehicle/sham groups, TNF-α immunoreactivity was distinctively observed in the CA1 pyramidal neurons (Figure 6A(a)). However, the TNF-α immunoreactivity in the vehicle/TFI group was increased at 1 day (about 115% vs. the vehicle/sham group) but significantly reduced at 5 days (about 38% vs. the vehicle/sham group) after TFI (Figure 6A(b,c),B).

In the CGA/sham group, TNF-α immunoreactivity in the CA1 pyramidal neurons was not different from that in the vehicle/sham group (Figure 6A(d),B). In addition, in the CGA/TFI group, the TNF-α immunoreactivity was not significantly altered at 1 day (about 96% vs. the vehicle/sham group) and 5 days (about 96% vs. the vehicle/sham group) after TFI (Figure 6A(e,f),B)).

#### 2.6.2. IL-2 Immunoreactivity

IL-2 immunoreactivity in the vehicle/sham group was easily shown in the CA1 pyramidal cells (Figure 6C(a)). In the vehicle/TFI group, however, the IL-2 immunoreactivity was increased at 1 day (about 118% vs. the vehicle/sham group); however, the IL-2 immunoreactivity was dramatically decreased at 5 days (about 61% vs. the vehicle/sham group) after TFI (Figure 6C(b,c),D).

In the CGA/sham group, IL-2 immunoreactivity in the CA1 pyramidal neurons was not different from that in the vehicle/sham group (Figure 6C(d),D). In the CGA/TFI group, the IL-2 immunoreactivity was not significantly changed at 1 day (about 98% vs. the vehicle/sham group) and 5 days (about 97% vs. the vehicle/sham group) after TFI (Figure 6C(e,f),D).

### 2.7. Anti-Inflammatory Cytokine Immunoreactivities in CA1 Pyramidal Cells

**Figure 7:**
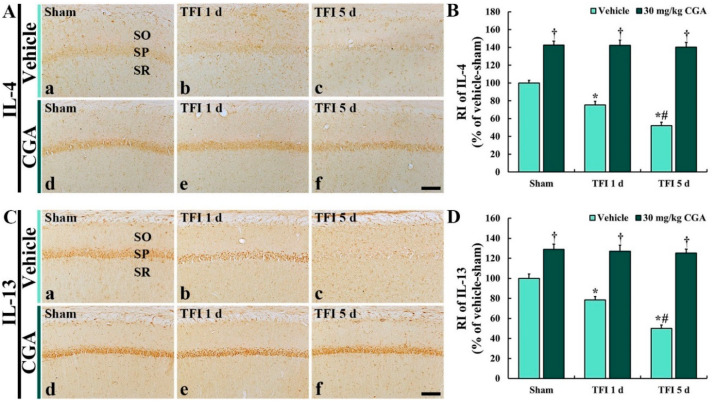
Immunohistochemistry for IL-4 (**A**) and IL-13 (**B**) of the vehicle/sham (**A**(**a**),**C**(**a**)), CGA/sham (**A**(**d**),**C**(**d**)), vehicle/TFI (**A**(**b**,**c**),**C**(**c**,**d**) and CGA/TFI (**A**(**e**,**f**),**C**(**e**,**f**) groups at 1 and 5 days after TFI. IL-4 and IL-13 immunoreactivities are detected in CA1 pyramidal cells of the vehicle/sham group. In the vehicle/TFI group, IL-4 and IL-13 immunoreactivities are gradually decreased after TFI. In the CGA/sham group, IL-4 and IL-13 immunoreactivities are markedly enhanced, and the increased immunoreactivities are maintained after TFI. Scale bar = 40 μm. C and D: RI of TNF-α (**C**) and IL-2 (**D**), respectively, in the CA1 SP. The bars indicate the means ± SEM (*n* = 7 at each point in time, * *p <* 0.05 vs. the vehicle/sham group, ^†^ *p* < 0.05 vs. the corresponding time point vehicle group, ^#^ *p <* 0.05 vs. the pretime point vehicle group).

Changes in anti-inflammatory cytokine immunoreactivities were investigated by immunohistochemistry for IL-4 and IL-13 in the CA1 region (Figure 7).

#### 2.7.1. IL-4 Immunoreactivity

In the vehicle/sham group, IL-4 immunoreactivity was clearly observed in the CA1 pyramidal cells (Figure 7A(a)). However, in the vehicle/TFI group, the IL-4 immunoreactivity was remarkably reduced at 1 day (about 75% vs. the vehicle/sham group) and more decreased at 5 days (about 51% vs. the vehicle/sham group) after TFI (Figure 7A(b,c),B).

In the CGA/sham group, IL-4 immunoreactivity in the CA1 pyramidal neurons was significantly higher (about 143% vs. the vehicle/sham group) than that in the vehicle/sham group (Figure 7A(d),B). Moreover, in the CGA/TFI group, the enhanced IL-4 immunoreactivity was maintained at 1 day (about 142% vs. the vehicle/sham group) and 5 days (about 140% vs. the vehicle/sham group) after TFI (Figure 7A(e,f),B).

#### 2.7.2. IL-13 Immunoreactivity

IL-13 immunoreactivity in the vehicle/sham group was also found in the CA1 pyramidal cells (Figure 7C(a)). However, the IL-13 immunoreactivity in the vehicle/TFI group was significantly decreased at 1 day (about 78% vs. the vehicle/sham group) and more reduced at 5 days (about 49% vs. the vehicle/sham group) after TFI (Figure 7C(b,c),D).

In the CGA/sham group, IL-13 immunoreactivity in the CA1 pyramidal neurons was significantly increased (about 129% vs. the vehicle/sham group) compared to that in the vehicle/sham group (Figure 7C(d),D). In the CGA/TFI group, the increased IL-13 immunoreactivity was sustained at 1 day (about 127% vs. the vehicle/sham group) and at 5 days (about 125% vs. the vehicle/sham group) after TFI (Figure 7C(e,f),D).

## 3. Discussion

It has been reported that pretreated *Oenanthe Javanica* extract rich in CGA exerts neuroprotective effects in the gerbil hippocampus following TFI via enhancement of antioxidant enzymes and regulation of anti- and pro-inflammatory cytokines [17,18]. In addition, Lu et al. (2019) reported that CGA is abundantly contained in *Oenanthe Javanica* [19]. Furthermore, it has been demonstrated that CGA and its metabolites, which exert a protective effect against an in vitro model of degenerative neuronal damage via antioxidant antioxidative activity mediated by proteasome inhibition [20], are closely involved in a neuroprotective pathway in neurodegenerative disorders [21]. Therefore, we used CGA to examine its neuroprotective potentiality and mechanisms in the hippocampus of a gerbil model of 5 min TFI.

The hippocampus is one of substructures vulnerable to ischemic insults, and the hippocampal injury is prone to declines in memory and learning [22,23]. In particular, these declines are easily shown in a gerbil model of TFI for 5 min, because this model gives rise to death of pyramidal neurons located in the SP of the hippocampal CA1 region about 4–5 days later [6,24,25,26]. In our current study, we found declines in learning and memory, and spatial memory in the vehicle/sham group following 5 min TFI. However, these impairments were alleviated by pretreatment with 30 mg/kg CGA. It has been demonstrated that neuroprotective materials display the attenuation of TFI-induced declines in cognitive function. For instance, the administration of Yokukansan, a traditional herbal medicine which is also known as “Kampo”, moderated declines in spatial memory in gerbils with 5 min TFI, showing that pyramidal neurons in the hippocampal CA1 region were protected against the ischemic injury [27].

Hermawati et al. (2020) investigated whether CGA treatment attenuated spatial memory decline by a Morris water maze test in rats subjected to transient ischemia induced by bilateral common carotid arteries occlusion (BCCAO) and found no significant differences in escape latency from the 3rd trial between the sham-operated and BCCAO-operated groups [14]. However, we examined not only spatial memory (by 8-ARMT) but also short-term memory test (by PAT) and found significant differences in the number of errors and latency time between the sham-operated group and TFI-operated group. In our opinion, the differences resulted from animal model of cerebral ischemia. The gerbils used in our current study have an incomplete circle of Willis in the base of the brain, in which posterior communicating arteries are lacking [28]. Therefore, in the gerbils, only ligation of both common carotid arteries leads to ischemia in the forebrain [29]. However, in the case of the rat model used by Hermawati et al. (2020), blood supply to the forebrain is not totally stopped when the rats were given BCCAO because of the presence of posterior communicating arteries in the Willis circle. In addition, many of the CA1 pyramidal neurons in the rat model (79.4% of the sham-operated rats) were found after BCCAO [14]. However, in our gerbil model, a few CA1 pyramidal neurons (10.2% of the sham group) were detected after TFI. Therefore, in our current study, CGA treatment displayed apparent effectiveness in the amelioration of TFI-mediated cognitive impairments.

It has been well acknowledged that neuronal loss/death following ischemic insults shows different patterns and degrees depending on complex ischemic conditions including kinds of occluded vessels, durations of ischemic period, body temperature, etc. For instance, we have reported that ischemia-mediated neuronal loss/death in the caudate–putamen is different in the pattern of neuronal death according to the subregions of the caudate–putamen and ischemic duration (15 and 30 min) in a rat model of transient focal brain ischemia induced by middle cerebral artery occlusion (MCAO) [3]. In addition, it has been reported that a severer aspect of neuronal loss/death in the hippocampus is shown following 15 min of TFI than that in 5 min TFI in gerbils [2]. Furthermore, a rat model of focal brain ischemia induced by MCAO for over 1 h develops infarct lesion (necrotic tissue) which is easily visualized by 2, 3, 5-triphenyltetrazolium chloride (TTC) assay, but infarction does not occur in the brain induced by TFI for 5–15 min in gerbils when the TTC assay is applied, in which selective neuronal death only occurs [1,15,30,31,32,33].

Based on those precedent studies, we used the gerbil model of 5 min TFI to study selective neuronal death and its mechanisms; animal models of MCAO for over 1 h do not provide selective neuronal death in the hippocampus because the MCAO does not impair the hippocampus. After the confirmation of the CA1 pyramidal cells in the ischemic gerbil hippocampus, we pretreated 7.5, 15 and 30 mg/kg CGA and found that 30 mg/kg CGA pretreatment protected the CA1 pyramidal cells (principal neurons in the hippocampal CA1 region) against 5 min TFI in the gerbil. CGA has been investigated to display neuroprotective potentiality against ischemic insults in various experimental animal models of cerebral ischemia. For example, Hermawati et al. (2020) described that 30 and 60 mg/kg CGA protected hippocampal pyramidal neurons following 20 min of TFI induced by occlusion of bilateral common carotid arteries in rats [14]. Additionally, Lee K et al. (2012) reported that 30 mg/kg CGA reduced infarct volume in the brain of a rat model of focal brain ischemia induced by middle cerebral artery occlusion for 2 h [34]. Taken together, the dose of 30 mg/kg CGA can display a neuroprotective effect following ischemia-reperfusion in rodent models of transient ischemia. In addition, 30 mg/kg of CGA can be applied in the clinical field as an optimal dose to protect brains against ischemic stroke.

It is well known that excessive production of ROS causes the denaturation of macromolecules (e.g., proteins, DNA, lipids, etc.) and ultimately causes cellular dysfunction [24,35,36]. In addition, the deficiency of ROS accompanies malfunctions in the immune system, which elevate the danger of bacterial spreading and trigger autoimmune disorder [37,38]. In this regard, the regulation of endogenous antioxidant enzymes including SOD1 and SOD2 plays essential roles in controlling redox homeostasis via well control between ROS generation and ROS scavenging [39,40]. In particular, the overproduction of ROS following ischemic insults is regarded as one of the major concerns leading to neuronal damage or death [8,9]. In order to qualify oxidative stress, there are two methods (direct and indirect methods). The direct method is to analyze the in situ production of superoxide anion by DHE fluorescence assay [11,41]. The indirect method is to quantify end-products generated by ROS via 4-HNE immunohistochemistry for lipid peroxidation [30,42]. In our current study, the excessive production of ROS and increased lipid peroxidation in the CA1 pyramidal cells was examined following 5 min TFI, whereas the pretreatment with 30 mg/kg CGA remarkably attenuated them, showing that SOD expressions were significantly increased in the CA1 pyramidal cells before and after TFI. Taken together, pretreated 30 mg/kg CGA can provide a neuroprotective effect via upregulation of antioxidant enzymes which scavenge overproduced ROS in the CA1 pyramidal neurons following TFI. This is supported by a number of studies that have demonstrated that increased antioxidant enzymes create neuronal resistibility against ischemia-reperfusion injury by attenuating oxidative stress [11,30,35,43,44].

Inflammatory responses are generally initiated by immunocytes that recognize surfaces of pathogens, such as bacteria and viruses [45,46]. In brains, however, resident microglia and/or immune cells, which are derived from the increase in blood–brain barrier permeability, are provoked in the brain under pathological conditions including ischemic stroke and neuroinflammation [47]. Since the brain is well known to be an aseptic organ, and pathogens are not involved in the neuroinflammatory response following ischemia-reperfusion injury, some studies call this response a “sterile inflammatory response” [46,48]. In addition, neuroinflammation is concerned with both beneficial and detrimental roles in ischemic brains, and these are modulated by anti- and pro-inflammatory cytokines [49,50,51]. It is well known that pro-inflammatory cytokines exacerbate and advance deleterious inflammatory processes, but anti-inflammatory cytokines suppress the expressions of pro-inflammatory cytokines and bring advantageous inflammatory processes in ischemic brains [10,52]. In this regard, many studies have reported that increases in anti-inflammatory cytokine expressions present neuroprotective effects following ischemia-reperfusion injuries [10,32,50,53]. In our current study, pro-inflammatory cytokines (TNF-α and IL-2) were increased in the CA1 pyramidal cells following TFI; however, pretreated 30 mg/kg CGA inhibited these increases and elevated the expression levels of anti-inflammatory cytokines (IL-4 and IL-13). These imply that pretreatment with 30 mg/kg CGA can increase anti-inflammatory cytokines and create a neuroprotective effect in the hippocampal CA1 region following TFI.

In conclusion, pretreatment with 30 mg/kg CGA revealed neuroprotective effects via antioxidative efficacy which was able to reduce oxidative stress and enhance antioxidant enzymes, and via anti-inflammatory efficacy which was able to down-regulate pro-inflammatory cytokines and improve anti-inflammatory cytokines. Taken together, we strongly suggest that CGA can be a useful neuroprotective material against ischemic injuries.

## 4. Materials and Methods

### 4.1. Experimental Animals and Protocol

Male Mongolian gerbils (6 months old; 80~90 g body weight) were supplied by the Experimental Animal Center of Kangwon National University (Chuncheon, Korea). The housing conditions for the animals were conventionally established under suitable room temperature (25 ± 2 °C) and relative humidity (about 50%). Freely accessible feed and water were provided to the gerbils, and steady dark/light cycles were controlled every 12 h.

The protocol of this study was sanctioned by Institutional Animal Care and Use Committee (IACUC) at Kangwon National University (approval no., KW-180124-1) and abided by the guidelines from the current international laws and policies in the “Guide for the Care and Use of Laboratory Animals” (The National Academies Press, 8th Ed., 2011).

### 4.2. Experimental Groups and CGA Treatment

Gerbils (total *n* = 100) were randomly divided into 8 groups as follows: (1) and (2) vehicle/sham (*n* = 12) and vehicle/TFI (*n* = 24) groups: each was treated with saline (0.85% *w*/*v* NaCl) as a vehicle and subjected to sham and TFI operation; (3) and (4) 7.5 mg/kg CGA/sham (*n* = 7) and CGA/TFI (*n* = 7) groups: each was pretreated with 7.5 mg/kg CGA and received sham and TFI operation; (5) and (6) 15 mg/kg CGA/sham (*n* = 7) and CGA/TFI (*n* = 7) groups: each was treated with 15 mg/kg CGA and given sham and TFI operation; (7) and (8) 30 mg/kg CGA/sham (*n* = 12) and CGA/TFI (*n* = 24) groups: each was treated with 30 mg/kg CGA and subjected to sham and TFI operation.

Both the vehicle and CGA were administered by intraperitoneal injection once a day for 5 days before TFI operation upon the method used by Miao et al. (2017) [13].

### 4.3. TFI Induction

In accordance with our published method, the surgical procedure of the induction of TFI in gerbils was performed [54]. In brief, using an inhaler, the gerbils were anesthetized with 2.5% isoflurane gas mixed with oxygen (33%) and nitrous oxide (67%) [55]. Under anesthesia, the ventral surface of the neck was shaved, made a midline incision, exposed common carotid arteries (CCA) on both sides and ligated with non-traumatic aneurysm clips for 5 min. During the ischemia, the stop of blood circulation to the brain was confirmed in the central artery of retinae with ophthalmoscope (HEINE K180^®^) (Heine, Optotechnik, Herrsching, Germany). After the conformation, the clips were removed, and the incised area was closed by using 3-0 suture silk (Ethicon Inc., Somerville, NJ, USA). During the surgical procedure, the body temperature was monitored in real time with a rectal temperature probe (TR-100) (Fine Science Tools, Foster City, CA, USA), and normothermic body temperature (37 ± 0.5 °C) was maintained. The surgical procedure of sham operation was identically carried out without ligation of the CCA.

### 4.4. Tests for Cognitive Functions

#### 4.4.1. 8-ARMT

To investigate the change in the spatial memory following TFI, 8-ARMT was conducted. According to a published protocol [27] with some modifications, in short, we utilized a maze that consisted of an opaque round acryl board (central platform; diameter, 20 cm) with radially extended eight arms (width, 6 cm; height, 10 cm; length, 25 cm) (Stoelting Co., Wood Dale, IL, USA). The gerbils in each group were trained once a day for three days before TFI. The substantive test was carried out daily for three days after TFI. We put a pellet feed at the end of each arm and each gerbil was placed onto the central platform of the maze. Each trial was terminated when the gerbil consumed all of the pellet feeds. We counted numbers of errors when the gerbil did not arrive at the end of the arm that the gerbil had already visited.

#### 4.4.2. PAT

To examine learning and memory after TFI, PAT was conducted according to a method [22] with some modifications. Briefly, the PAT was carried out with the Gemini Avoidance System (GEM 392; San Diego Instruments, San Diego, CA, USA). The system was divided by a vertical gate into light and dark compartments. The gerbils in each group were trained for three days before TFI as follows. Each gerbil was allowed to explore both light and dark compartments for three minutes. The vertical gate was shut down followed by giving a foot-shock (0.5 mA for 5 s) as soon as the gerbil entered the dark compartment. Five days after TFI, substantive PAT was performed as follows. Each gerbil was put into the light compartment, and the vertical gate was opened. Latency time was recorded until the gerbil went into the dark compartment. Maximum latency time to stay in the light compartment was designated as 180 s.

### 4.5. Western Blotting

To examine the effects of pretreated CGA on the expressions of antioxidant enzymes and anti-inflammatory cytokines in the hippocampal CA1 region which contain pyramidal neurons following TFI, Western blotting was carried out as previously described [10,35]. In short, gerbils were deeply anesthetized via intraperitoneal injection of 60 mg/kg pentobarbital sodium at sham, 1 day and 4 days after TFI, and their brains were removed [55]. Brain tissues containing the hippocampus were dissected, and they were serially and transversely cut into 400 μm thickness with a vibratome. Hippocampal CA1 regions were harvested with a surgical blade under an enlarger. These were homogenized in 50 mM PBS (pH 7.4) containing ethylene glycol-bis (2-aminoethyl ether)-tetraacetic acid (pH 8.0), 0.2% Nonidet P-40, 10 mM ethylenediaminetetraacetic acid (pH 8.0), 15 mM sodium pyrophosphate, 150 mM sodium chloride, 100 mM β-glycerophosphate, 50 mM sodium fluoride, 2 mM sodium orthovanadate, 1 mM phenylmethanesulfonyl fluoride and 1 mM dithiothreitol. The homogenized tissues were centrifuged, and the protein level in the supernatants was determined with a Micro bicinchoninic acid protein assay kit with bovine serum albumin as a standard (Pierce Chemical, Dallas, TX, USA). The aliquots containing 50 μg of total protein were boiled in loading buffer containing 250 mM Tris (pH 6.8), 10 mM DTT, 10% sodium dodecyl sulfate, 0.5% bromophenol blue and 50% glycerol and subsequently loaded onto 5% polyacrylamide gel. After electrophoresis, the gels were transferred to nitrocellulose membranes (Pall Crop, East Hills, NY, USA). To be immunoreacted with each antibody, the same membrane stripes were used. The membranes were incubated to lessen background staining with 5% skimmed milk in TBS containing 0.1% Tween 20 and then immunoreacted with each primary antibody. Primary antibodies were as follows: rabbit anti-SOD2 (1:2000, Abcam, Cambridge, UK), goat anti-4-HNE (1:5000, Abcam, Cambridge, UK), goat anti-IL-4 (1:500, Santa Cruz Biotechnology, Santa Cruz, CA, USA), rabbit anti-IL-2 (1:500, Santa Cruz Biotechnology, Santa Cruz, CA, USA) and rabbit anti-β-actin (1:2000, Sigma-Aldrich, St. Louis, MO, USA). Each immunoreaction was conducted overnight, and the aliquots were incubated with peroxidase conjugated secondary antibodies: goat anti-rabbit IgG (1:4000, Santa Cruz Biotechnology, Santa Cruz, CA, USA) and donkey anti-rabbit IgG (1:5000, Santa Cruz Biotechnology, Santa Cruz, CA, USA). Finally, a luminol-based chemiluminescence kit (Pierce; Thermo Fisher Scientific Inc., Waltham, MA, USA) was used for enhancement of visualization.

The analysis of the blots was performed as previously described [35]. In short, the bands were scanned and densitometric analysis was conducted to quantify the bands with Scion Image software (Scion Crop., Frederick, MD, USA). Each level of the target protein was normalized via the corresponding level of β-actin.

### 4.6. Tissue Preparation for Histological Examination

Preparation of hippocampal tissue sections was carried out as previously described [54]. In short, the gerbils were deeply anesthetized by intraperitoneal injection of 60 mg/kg of pentobarbital sodium (JW pharm. Co., Ltd., Seoul, Korea) [55]. Under deep anesthesia, the brains of these animals were rinsed by perfusion (flow rate, 6 mL/min; total perfused volume, 60 mL) with 100 mM phosphate-buffered saline (PBS, pH 7.4) via the ascending aorta and fixed then with a solution of 4% paraformaldehyde (in 100 mM PB, pH 7.4) for fixation. The brains were harvested and post-fixed with the same fixative for 6 h at room temperature. For cryoprotection, the fixed brains were infiltrated in 30% sucrose solution (in 100 mM PB, pH 7.4). Finally, these brains were serially and coronally sectioned into 30 μm thicknesses in cryostat (Leica, Nussloch, Germany)

### 4.7. F-J B Histofluorescence Staining

To investigate the protective effect of CGA in the hippocampal CA1 region at 5 days after TFI, F-J B histofluorescence staining was carried out as described previously [54]. Briefly, the prepared sections were reacted with 0.06% potassium permanganate (KMnO_4_) solution (in distilled water) for 20 min at room temperature. Next, they were stained in 0.0004% F-J B (Histochem, Jefferson, AR, USA) solution for 40 min at room temperature. These stained sections, subsequently, were dehydrated and mounted with cover glasses using dibutylphthalate polystyrene xylene (DPX) (Sigma, St. Louis, MO, USA) as a mounting medium.

To observe the fluorescence stained by F-J B, we used an epifluorescent microscope (Carl Zeiss, Oberkochen, Germany) equipped with 450–490 nm blue excitation light coupled with a camera (DP72, Olympus, Japan). As described previously [35], seven sections per gerbil were selected to quantitatively analyze for F-J B^+^ cells. Using the epifluorescent microscope, the digital images of the F-J B^+^ cells were captured. Finally, the F-J B^+^ cells were counted in 250 μm × 250 μm^2^ in the CA1 region with Using Optimas 6.5 software (CyberMetrics, Phoenix, AZ, USA).

### 4.8. DHE Histofluorescence Staining

Fluorescent DHE assay was conducted to examine the in situ production of superoxide anion in the CA1 region. In brief, according to our published method [35], the sections were equilibrated in Krebs-HEPES buffer (pH 7.4) containing 5.6 mM KCl, 2 mM CaCl2, 0.24 mM MgCl2, 130 mM NaCl, 8.3mM HEPES and 11 mM glucose for 30 min at 37 °C. After then, the sections were immersed into fresh buffer containing 0.01 mM DHE (Sigma-Aldrich, St. Louis, MO, USA) for 2 h at 37 °C. DHE was oxidized in the reaction with superoxide to ethidium which binds with DNA in nuclei and fluoresced in red.

The in situ production of superoxide anion was analyzed as previously described [11]. Briefly, seven sections/gerbil were chosen in order to analyze the fluorescence intensity. Images of DHE fluorescence were captured with an epifluorescent microscope (Carl Zeiss, Germany) equipped with 520–540 nm of excitation wavelength. Finally, ethidium fluorescence was quantified from the captured images using Image-pro Plus 6.0 software. The ratio of the DHE fluorescence intensity was calibrated as a percentage.

### 4.9. Immunohistochemistry

To investigate the neuroprotective effect by CGA and its protective mechanisms, immunohistochemistry was performed in accordance with our previously published method [30]. In short, the brain sections were immersed in 0.3% hydrogen peroxide (H_2_O_2_, in 10 mM PBS, pH 7.4) solution for 30 min at room temperature. Next, these were reacted with 10% normal horse or goat serum solution (in 10 mM PBS, pH 7.4) for 40 min at room temperature. After, they were incubated with each primary antibody for 12 h at 4 °C. Primary antibodies were as follows: mouse anti-NeuN (1:800, Chemicon, Temecula, CA, USA) to investigate neurons; mouse anti-4-HNE (1:1000, Alexis Biochemicals, San Diego, CA, USA) to examine oxidative stress; sheep anti-SOD1 (1:1000, Calbiochem, La Jolla, CA, USA) and sheep anti-SOD2 (1:1000, Calbiochem, La Jolla, CA, USA) to investigate antioxidant enzymes; rabbit anti-TNF-α (1:1000, Abcam, UK) and rabbit anti-IL-2 (1:200, Santa Cruz Biotechnology, Santa Cruz, CA, USA) to examine pro-inflammatory cytokines; mouse anti-IL-4 (1:250, Santa Cruz Biotechnology, Santa Cruz, CA, USA) and rabbit anti-IL-13 (1:250, Santa Cruz Biotechnology, Santa Cruz, CA, USA) to examine anti-inflammatory cytokines. The sections immunoreacted with each primary antibody were, subsequently, immersed in biotinylated secondary antibodies: horse anti-mouse IgG and goat anti-rabbit or sheep IgG (1:250, Vector, Burlingame, CA, USA) for 2 h at room temperature. These, thereafter, were exposed to avidin–biotin complex (ABC, 1:300, Vector, Burlingame, CA, USA) for 1 h at room temperature. Finally, they were visualized by reacting with 3, 3′-diaminobenzidine tetrahydrochloride (Sigma, St. Louis, MO, USA) solution (in 100 mM PBS, pH 7.4).

As previously described [2,10,35], corresponding immunoreactive structures were analyzed in the CA1 region. Seven sections/gerbil were selected. First, for the quantitative analysis of NeuN+ cells, their images were captured with an optical microscope (BX53) (Olympus, Japan) equipped with a camera (DP72) (Olympus, Japan). Cell count was performed in 250 μm × 250 μm^2^ in the CA1 region by using Optimas 6.5 software (CyberMetrics, Phoenix, AZ, USA). Second, for quantitative analyses of SOD1, SOD2, 4-HNE, IL-4, IL-13, TNF-α and IL-2 immunoreactivity, digital images of each immunoreactive structure were captured. These images were calibrated into an array of 512 × 512 pixels in 140 × 140 μm^2^. Individual immunoreactivity was quantified by a 0–255 gray scale system after the background noise was subtracted. Relative immunoreactivity (RI), as a ratio of the immunoreactivity, was presented by using Adobe Photoshop (version 8.0) (Adobe, San Jose, CA, USA) and Image J software (version 1.59; National Institutes of Health, Bethesda, MD, USA).

### 4.10. Statistical Analysis

Data obtained in this experiment were presented as the mean ± standard error of the mean (SEM). Statistically, the data were measured using SPSS 18.0 software (SPSS, Chicago, IL, USA). Two-way analysis of variance (ANOVA) with a post hoc Bonferroni’s test was applied to establish significant differences among the experimental groups. At under 0.05 of P value, statistical significance was designated.
